# A method for reducing the sloughing of thick blood films for malaria diagnosis

**DOI:** 10.1186/1475-2875-12-231

**Published:** 2013-07-08

**Authors:** Andrew P Norgan, Heather E Arguello, Lynne M Sloan, Emily C Fernholz, Bobbi S Pritt

**Affiliations:** 1 Department of Medicine, Mayo Clinic, Rochester, MN, USA; 2 Division of Clinical Microbiology, Mayo Clinic, Rochester, MN, USA

**Keywords:** Malaria, Thick films, Adherence, Sloughing

## Abstract

**Background:**

The gold standard for malaria diagnosis is the examination of thick and thin blood films. Thick films contain 10 to 20 times more blood than thin films, correspondingly providing increased sensitivity for malaria screening. A potential complication of thick film preparations is sloughing of the blood droplet from the slide during staining or rinsing, resulting in the loss of sample. In this work, two methods for improving thick film slide adherence (‘scratch’ (SCM) and ‘acetone dip’ (ADM) methods) were compared to the ‘standard method’ (SM) of thick film preparation.

**Methods:**

Standardized blood droplets from 26 previously examined EDTA whole blood specimens (22 positive and four negative) were concurrently spread on glass slides using the SM, ADM, and SCM. For the SM and ADM prepared slides, the droplet was gently spread to an approximate 22 millimeters in diameter spot on the slide using the edge of a second glass slide. For the SCM, the droplet was spread by carefully grinding (or scratching) it into the slide with the point of a second glass slide. Slides were dried for one hour in a laminar flow hood. For the ADM, slides were dipped once in an acetone filled Coplin jar and allowed to air dry. All slides were then Giemsa-stained and examined in a blinded manner. Adherence was assessed by blinded reviewers.

**Results:**

No significant or severe defects were observed for slides prepared with the SCM. In contrast, 8 slides prepared by the ADM and 3 prepared using the SM displayed significant or severe defects. Thick films prepared by the three methods were microscopically indistinguishable and concordant results (positive or negative) were obtained for the three methods. Estimated parasitaemia of the blood samples ranged from 25 to 429,169 parasites/μL of blood.

**Conclusions:**

The SCM is an inexpensive, rapid, and simple method that improves the adherence of thick blood films to standard glass slides without altering general slide preparation, microscopic appearance or interpretability. Using the SCM, thick films can be reliably examined less than two hours after sample receipt. This represents a significant diagnostic improvement over protocols requiring extended drying periods.

## Background

Although rapid diagnostic and molecular tests for malaria are increasing in prevalence and importance, the standard method for malaria diagnosis in much of the world remains the examination of thick and thin blood films [1]. Microscopy, while laborious and technically challenging, persists through its combined advantages of cost, availability, and relative sensitivity. The standard malaria blood films are the thin (an unlysed fixed sheet of individual blood cells) and the thick film (a multilayered film of unfixed lysed blood) [1]. First described by Ross in 1903 (although used by Ross and Leishman for years prior), the thick film is estimated to be 10–20 times more sensitive than the thin film, with a detection threshold under ideal conditions of 10–50 parasites/μL of blood [2-5]. The sensitivity of malaria blood films, and in particular thick films, can vary greatly with preparation methods, experience and skill of the microscopist, and time spent examining the film. Under non-ideal field lab conditions, the estimated sensitivity is only 100-500 parasites/uL, which is comparable to the antigen-detection methods under similar conditions [4-6]. Unfortunately, a complication of thick film preparation in both laboratory and field settings is sloughing of some or the entire blood droplet during preparation, usually during the post-staining rinse [2,7-13]. Loss of sample during preparation may necessitate preparing a replacement slide, if additional sample blood is available, and delay diagnosis. A number of suggestions for ameliorating blood droplet sloughing can be found in a review of the literature, including: 1) extended blood droplet drying times (up to 24 hours) [3,12,13]; 2) utilization of an oven or heatblock to dry the sample [3,9,14-16]; 3) brief washes in methanol or acetone to help dehydrate the sample [8,10,11]; and 4) use of very small blood volumes (<5 μL) to prepare the thick film in conjunction with an alternative staining procedure [16].

Each of these suggested modifications in technique have potential advantages and disadvantages. For instance, extending the drying time of the thick film beyond the minimum necessary time is a simple and cost effective method that may improve adherence of the droplet, but it works directly counter to the idea of blood film examination as a rapid diagnostic procedure and leads unavoidably to a delay in diagnosis (and potentially treatment) if parasitaemia is not evident by examination of concurrently prepared thin film slides.

By contrast, use of a heatblock or oven to aid in drying thick films is an inexpensive way to both improve blood droplet adherence and speed thick film preparation, albeit with a few obvious disadvantages. A notable complication of heating thick films is that fixation of red cells can occur if either the temperature or the duration of heating are not carefully controlled; for this reason, it is not widely recommended [8]. In contrast to the thin blood film, the thick film cannot be fixed before staining, as fixation would prevent osmotic lysis and dehaemoglobinization. Interestingly, there are several references in the literature to either methanol or acetone washes which are used to quickly dehydrate and partially ‘fix’ the thick blood films [8]. It is not clear if this technique has been widely used, but a commonly used parasitology laboratory reference suggests that quick dips in acetone (no more than two) may improve the adherence of thick films [8]. Smrkovski and colleagues have reported that a dip in methanol followed by a dip in acetone reduces sloughing [11]. In contrast, Iqbal *et al.* have found that incorporation of three acetone dips in a rapid Giemsa staining protocol led to poorer droplet adherence than a standard Giemsa staining technique without acetone [10].

It has been noted that a larger blood droplet contributes to the tendency of a thick film to slough from the slide. Thellier *et al.* addressed this problem directly by recommending that <5 μL of blood be used in their rapid staining method [16]. This procedure appears promising, as the authors reported increased adherence, increased speed of analysis, and improved sensitivity. However, it may not be easily implemented in all settings due to requirements for additional reagents (e.g., saponin) and precise control of blood droplet volume [16].

A standard practice in the authors’ clinical laboratory has been to prepare malaria thick films by grinding or ‘scratching’ the blood droplet into the slide with a second glass slide in order to reduce sloughing of the blood droplet during slide preparation. This technique has not been described or compared to standard method (SM) in the literature, and therefore the efficacy of the scratch method (SCM) in improving the adherence of blood droplets as compared to SM or an acetone dip (ADM) was examined.

## Methods

### Slide preparation

Standardized blood droplets (35 μL) from twenty-six previously examined EDTA whole blood specimens (22 positive and 4 negative) were placed on glass slides in triplicate to prepare Giemsa-stained thick films by either ‘Standard’, ‘Acetone Dip’ and ‘Scratch’ methods (SM, ADM and SCM, respectively). For the SM and ADM prepared slides, the droplet was gently spread to an approximate nickel-sized area (~22 mm in diameter) on the slide using the edge of a second glass slide. For the SCM, the droplet was spread by carefully grinding (or scratching) it with moderate force into the slide with the point of a second glass slide. All slides were dried for one hour in a laminar flow hood. For the ADM, slides were dipped once in an acetone filled Coplin jar and allowed to air dry. Slides were Giemsa-stained using established protocols [8] and examined in a blinded manner for parasite identification by three independent trained examiners. Positive and negative parasite identification represents either complete agreement (24 cases) or the consensus of two reviewers (2 cases). Percent parasitaemia was calculated from the SCM slide in each three-slide set by concurrently counting parasites and leukocytes until two hundred were counted (or five hundred if less than ten parasites were counted with the first two hundred). Parasite density calculated using an estimated density of 8,000 leukocytes per microliter of blood in order to demonstrate the approximate range of parasitaemia in the positive blood specimens used in this study.

### Adherence assessment

Adherence was assessed by 3 blinded reviewers using the following scale: 3 - no defect (90-100% sample remaining), 2 - minor defect (66-89% sample remaining), 1 - significant defect (11-65% sample remaining), 0 - severe defect (10% or less sample remaining). Three blinded reviewers independently estimated the severity of the defects assessed sample defects using the indicated scale. The assigned adherence scores represent either a complete agreement (18 cases) or the consensus of two reviewers (eight cases).

## Results

### Blood droplet adherence

**Figure 1:**
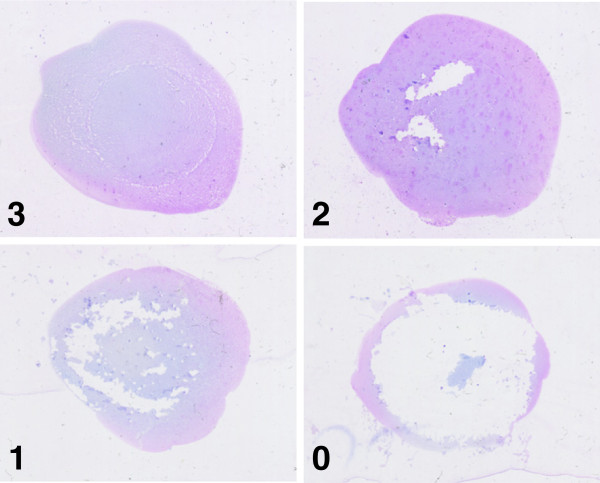
**Assessment of blood droplet adherence.** Examples of different levels of blood droplet adherence and associated adherence scores.

**Figure 2:**
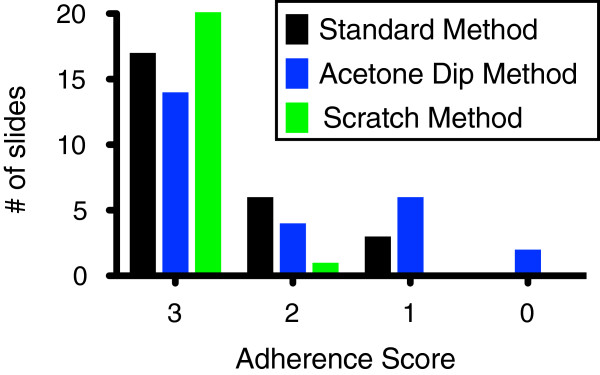
**Frequency distribution of adherence scores of the blood droplets by method.** Slides prepared by the scratch method had the highest frequency of complete adherence and did not have any instances of significant defects (score 1) or severe defects (score 0). The acetone dip did not improve droplet adherence over the standard preparation method.

An adherence score was assigned to each slide in blinded fashion (for representative images, see Figure 1). Slides prepared with SCM displayed both a lower frequency and a lesser severity of blood droplet adherence defects than slides prepared by the SM or ADM (Figure 2). There were no instances of significant defects (1 on adherence scale) or severe defects (0 on adherence scale) observed for scratched slides (Figure 2). ADM prepared slides did not perform as well as the SCM or SM in terms of frequency or severity of adherence defects (Figure 2). For the SCM the most frequently observed result was a completely adherent blood droplet (25/26), with a single minor adherence defect. While completely adherent droplets were also the most common result for SM or ADM prepared slides, the incidence of adherence defects was significantly higher SM (9/26) or ADM (12/26) (Figure 2).

### Thick film appearance and interpretation

The appearance of thick films prepared by the three preparation methods were considered microscopically indistinguishable (for the two slides with severe sample loss assessment was based on the small portion of remaining sample), with no artifacts noted with either the SCM or ADM. Concordant positive or negative results were obtained for slides prepared by the three methods. Despite severe adherence defects in some slides, it was still possible to assess positivity in the 22 positive slides. Similarly, there was agreement for the 4 negative cases (i.e., no artifacts that complicated interpretation were introduced by any of the techniques). Estimated parasitaemia ranged from 25 to 429,169 parasites/μL of blood.

## Discussion

Scratching the slides while preparing malaria thick films has been the standard practice in the authors’ clinical parasitology laboratory for over 40 years. The origins of this practice are not known, but it has been believed to improve the adherence of thick blood films to slides, allowing for drying times of less than 30 minutes with little or no loss of the blood film. The method is simple to perform and uses only moderate force to scratch the slide with the point of another slide, thus posing no biosafety risk to laboratory personnel.

The recommendations for sample volume in malaria thick films vary by source and generally fall within the range of 5–40 μL [16-21]. Given these ranges and the standard practice of the authors’ laboratory, a droplet volume of 35 μL was used in this study. A 35 μL volume is consistent with the recommendations of the Clinical Microbiology Procedures Handbook and is also a droplet volume likely to be obtained when using unmeasured blood droplets (e.g., from a disposable transfer pipette or with blood collection via finger prick) [21].

The results confirm that the SCM slides were universally free of significant defects and without alteration of either appearance or interpretative quality. In contrast, approximately one-third of slides prepared by the standard method displayed some defect in blood droplet adherence after standard Giemsa staining.

In contrast to some previous reports, no adherence benefit from immersion in acetone was observed in this study. A potential caveat of this result is that only a single immersion was performed in this study, while two or three dips have been reported in other studies [8,10]. Another possibility is that acetone fixation is more effective with smaller volume blood droplets than used in this work. Of note, Iqbal *et al.* have also reported poorer adherence in acetone washed samples compared to slides prepared by a standard method. Unfortunately, differences in the staining procedures used in that study prevent ascribing adherence differences to the use of acetone washes alone.

It is important to note that the scratch method improves droplet adherence in the conditions used in this study (a temperature and humidity controlled laboratory using new glass slides), but factors such as temperature, humidity, slide cleanliness, and sample hematocrit may independently modulate droplet adherence. This work did not directly address those variables, and although samples did possess varying hematocrit it was not a controlled variable in the study. It is unknown if the scratch technique will be useful in settings where slides are cleaned and reused. The SCM is destructive to the slide in that grooves are etched into the glass when scratching. It is likely that repeatedly etching the slides may eventually lead to an undesirable diminution of slide transparency. However, it may not be necessary to repeatedly etch the slides if the blood droplet adherence benefits are similar with slides that have been ‘pre-etched’.

## Conclusions

The scratch method is an inexpensive, rapid, and simple method that improves the adherence of thick blood films to standard glass slides without altering slide preparation, microscopic appearance or interpretability. Using the scratch method thick films can reliably be examined less than two hours after sample receipt. This represents a diagnostic improvement over protocols requiring extended drying periods to ensure droplet adherence.

## Abbreviations

SCM: Scratch method; ADM: Acetone dip method; SM: Standard method.

## Competing interests

The authors declare no competing interests.

## Authors’ contributions

Conceived and designed the experiments: APN, BSP. Performed the experiments: APN, HEA, LMS, ECF, BSP. Analyzed the data: APN, BSP. Contributed reagents/materials/analysis tools: HEA, LMS, ECF. Wrote the paper: APN, BSP. All authors approved the final version of this manuscript.
